# Outgroup emotion processing in the vACC is modulated by childhood trauma and CACNA1C risk variant

**DOI:** 10.1093/scan/nsy004

**Published:** 2018-01-29

**Authors:** Johannes T Krautheim, Benjamin Straube, Udo Dannlowski, Martin Pyka, Henriette Schneider-Hassloff, Rebecca Drexler, Axel Krug, Jens Sommer, Marcella Rietschel, Stephanie H Witt, Tilo Kircher

**Affiliations:** 1 Department of Psychiatry and Psychotherapy, Philipps-University Marburg, 35039 Marburg, Germany; 2 Department of Psychiatry and Psychotherapy, University of Münster, 48149 Münster, Germany,; 3 Department of Genetic Epidemiology in Psychiatry, Central Institute of Mental Health, Medical Faculty Mannheim, Heidelberg University, 68159 Mannheim, Germany

**Keywords:** anterior cingulate cortex, minimal group paradigm, outgroup, environment, genetics

## Abstract

A high frequency of outgroup contact—as experienced by urban dwellers and migrants—possibly increases schizophrenia risk. This risk might be further amplified by genetic and environmental risk factors, such as the A-allele of rs1006737 within the calcium voltage-gated channel subunit alpha1 C gene and childhood interpersonal trauma (CIT). Both have been related to ventral anterior cingulate cortex (vACC) functioning. We investigated vACC functioning, during ingroup and outgroup emotion perception in relation to rs1006737 and CIT. Group membership was manipulated through a minimal group paradigm. Thus, in our functional magnetic resonance imaging study, a group of healthy Caucasian participants (*n* = 178) viewed video-recorded facial emotions (happy *vs* angry) of actors artificially assigned to represent the ingroup or the outgroup. Rs1006737 and CIT were related to brain activation for group and emotion specific processing. The group–emotion interaction in the vACC showed reduced sensitivity to emotional valence for outgroup member processing. Specifically for the angry outgroup condition, we found a gene by environment interaction in vACC activity. We speculate that the increased schizophrenia risk in migrants and urban dwellers could therefore be facilitated via this pathophysiological pathway.

## Introduction

Urban dwellers and migrants live in highly complex social structures with equally complex intergroup relations. Both have an increased risk for schizophrenia (Cantor-Graae and Selten, 2005; Das-Munshi *et al.*, 2012; Vassos *et al.*, 2012). Minority migrant groups experience increased psychosis risk when they stand out from their social environment, for example, through a different skin colour (Akdeniz *et al.*, 2014a), particularly when living in neighborhoods where own-group density is lower (Das-Munshi *et al.*, 2012), implicating a relevance of outgroup confrontation in this context. Especially humans with genetic and other environmental risks, such as the A-allele of rs1006737 within the calcium voltage-gated channel subunit alpha1 C (CACNA1C) gene and childhood interpersonal trauma (CIT) might be sensitive to frequent outgroup confrontation. In this context, the ventral anterior cingulate cortex (vACC) has been suggested to be an important converging zone of genetic and environmental risk factors for schizophrenia (Tost and Meyer-Lindenberg, 2012; Akdeniz *et al.*, 2014a). Indeed, alterations in vACC structure or functioning have been associated not only with ethnic-minority status (Akdeniz *et al.*, 2014b) and urban upbringing (Lederbogen *et al.*, 2011) but also with ingroup/outgroup processing (Volz and Kessler, 2009), the risk allele at rs1006737 within the CACNA1C gene (Wang *et al.*, 2011), and experience of CIT (Van Harmelen *et al.*, 2010). However, evidence about the exact interplay between rs1006737 and CIT regarding ingroup/outgroup processing is missing.

Numerous behavioral studies have shown the differential cognitive and emotional processing of ingroup and outgroup members, i.e. the misperception of emotional facial expressions in members of a different race (Elfenbein and Ambady, 2002), the misperception of non-verbal cues (gaze, posture gestures) (Hugenberg and Bodenhausen, 2004; Dovidio *et al* ., 2006) and difficulties with theory of mind tasks (Adams *et al.*, 2010). However, the cognitive processing and correlated brain activation for the membership of groups delineated by sex or race are liable to uncontrolled effects, such as emotional stereotypes, which are independent of a ‘pure’ group effect (Wheeler and Fiske, 2005). Hence, it is impossible to differentiate the effect of prejudice or stereotypes from pure effect of group membership when the group is modulated by ethnicity. Studies on the effects of group membership using different ethnicities are confronted with this bias (Cikara and Van Bavel, 2014). To circumvent this bias, ingroup *vs* outgroup classification can be achieved using the well-established minimal group paradigm (MGP), creating artificial ad hoc groups (e.g. using two different coloured arm bands; Taijfel, 1970; Tajfel *et al.*, 1971; Cikara and Van Bavel, 2014). MGP effects on behavior have been demonstrated widely in social psychology experiments (Tajfel *et al.*, 1971; Diehl, 1990). These advantages make the MGP especially suitable for investigating the association of neural ingroup/outgroup processing with genetic and environmental risk factors for schizophrenia, such as rs1006737 and CIT. The neural correlates of ingroup/outgroup membership have been demonstrated using functional magnetic resonance imaging (fMRI), which has shown a relevance of the ACC and the medial prefrontal cortex in the processing of racial (Eres and Molenberghs, 2013; Cikara and Van Bavel, 2014) and minimal group membership (Volz and Kessler, 2009). The latter study of Volz and colleagues was the first who used fMRI to investigate neural processing in group-related tasks based on a modified version of the MGP. In this study, participants distributed money between ingroup members (ingroup trial), between outgroup members (outgroup trial) or between ingroup and outgroup members (mixed trial). Comparative intergroup evaluation processes (mixed group trials *vs* same group trials) elucidated a network including the vACC, dorsomedial prefrontal cortex, temporoparietal junction and precuneus. Moreover, the vACC is part of a facial emotion processing network, which also processes happy and angry facial expressions (Fusar-Poli *et al.*, 2013). Yet, the influence of group membership on neural activation in the vACC for emotional facial expressions has not been investigated, and the relevance of genetic and environmental factors in this context is unknown.

*CACNA1C* has been repeatedly identified as a susceptibility gene for bipolar disorder, major depressive disorder, schizophrenia and schizotypal traits (Ferreira *et al.*, 2008; Sklar *et al.*, 2008; Green *et al.*, 2010; Nyegaard *et al.*, 2010; Roussos *et al.*, 2013; Ripke *et al.*, 2014, 2013; Takahashi *et al.*, 2015; Rao *et al.*, 2016; Starnawska *et al.*, 2016). *CACNA1C* encodes the pore-forming alpha-1c sub-unit of the L-type voltage-gated calcium channel and plays an important role in dendritic development, synaptic plasticity, learning and memory formation (Bhat *et al.*, 2012). Rs1006737 risk variant carriers have been shown to demonstrate vACC dysfunction while performing episodic memory tasks that correlates with activation patterns in first-degree relatives of patients with bipolar disorder, major depression or schizophrenia (Erk *et al.*, 2014). Neural activation for facial emotion perception in the vACC is altered in patients with schizophrenia (Taylor *et al.*, 2012). However, the influence of rs1006737 on neural vACC activation for facial emotion processing in the interplay with environmental risk factors remains unknown. CIT is an epidemiologically validated environmental risk factor for schizophrenia and affective disorders (Gilbert *et al.*, 2009; Matheson *et al.*, 2012) and is associated with hyper-reactivity of the amygdala during the presentation of threat-related faces, implicating a dysfunction of the regulatory effect of prefrontal areas on limbic structures (Dannlowski *et al.*, 2012). In line with this assumption, the experience of CIT is associated with reduced vACC grey matter volume (Van Harmelen *et al.*, 2010; Dannlowski *et al.*, 2012) and an impaired emotional regulation during the perception of negative emotional stimuli (New *et al.*, 2009).

In summary, ingroup/outgroup perception—being related to migration and urban upbringing—and the schizophrenia risk factors rs1006737 and CIT are associated with structural and functional alterations in the vACC. Although suspected, no study has yet investigated how genetic and environmental factors converge in the vACC in this specific context.

In this study, we investigated the neural correlates of group-dependent (ingroup *vs* outgroup) facial emotion processing and their relation to rs1006737 and CIT. Therefore, we used a MGP in an autochthonous German sample. We hypothesized (1) an interaction of neural activation for the processing of emotional valence (happy *vs* angry) and group membership (ingroup *vs* outgroup) in the vACC. We further hypothesized (2) a moderation of vACC function in response to group-dependent (ingroup *vs* outgroup) emotion processing by rs1006767 and CIT.

## Materials and methods

### Subjects

Study participants were 198 healthy, right-handed, Caucasian German native speakers of central European decent aged 19–39 years (M: 24.04 years; SD: 3.25 years, 50% males). We excluded 18 subjects due to head movement exceeding one voxel size (3.6 mm) during fMRI scanning, one subject due to insufficient engagement in post-scanning behavioural tasks, and one because of an incidental finding of a brain pathology leaving 178 subjects for the final analysis. One participant was excluded from genetic analysis due to a missing blood sample. Our study was approved by the local ethics committee, according to the Declaration of Helsinki, and all participants gave written informed consent prior to the commencement of the study.

### DNA extraction and genotyping

Genomic DNA was prepared from whole blood samples according to standard procedures. Rs1006737 was genotyped using a TaqMan 5' nuclease assay. Accuracy was assessed by duplicating 15% of the sample, and reproducibility was 100%. Fifteen subjects were rs1006737 homozygous A, while 91 were heterozygous GA, and 71 were homozygous G. The allele frequencies did not deviate from the Hardy–Weinberg equilibrium (*P* = 0.23). Genetic risk was defined by A-allele carrier status for rs1006737 (AA/AG vs. GG).

### Measuring childhood trauma and schizotypal traits

Childhood trauma was measured via the Trauma History Questionnaire (THQ) (Green, 1996). This 27-item self-report instrument determines possible exposure to a wide range of traumata. We focused on traumatic events with an interpersonal-interaction component such as being a victim of robbery or of sexual or physical abuse (10 items). Our sample was divided into groups of those with at least one self-reported traumatic event and those with no traumatic events before the age of 18. Schizotypal traits were assessed using the Schizotypal Personality Questionnaire-Brief (SPQ-B), a 22-item instrument with adequate reliability and validity (Raine and Benishay, 1995).

### Behavioural data analysis

All non-fMRI data were analysed using SPSS 20.0.0 for Windows (SPSS, Chicago, IL). The interaction of CIT and the rs1006737 risk allele was tested, as a proof of concept, regarding schizotypal traits as a proxy for schizophrenia risk (Vollema *et al.*, 2002) using general estimation equations (GEE) with a multi-factorial design.

### fMRI task and procedure

#### MGP manipulation

In order to create ad-hoc minimal groups, subjects completed a false psychological test where they answered questions regarding personality and problem-solving strategies. They were told that the purpose of this test was to identify strategies for solving a problem. After completing the test, which took about 5–10 min, participants were told that they were either a ‘conclusive’ problem solver with a ‘holistic approach’ or a ‘sequential’ problem solver with an ‘analytical approach’. In fact, these problem-solver types do not exist, and subjects had been randomly assigned to one of these groups before the experiment. To increase group identification, the participants had to read a short text with a further description of the two ‘personality types’. Before and after the fMRI session, participants were asked to indicate the degree of identification with the own problem-solver type (ingroup) and the other problem-solver type (outgroup) on a computer screen, which was assessed on a 7-point Likert scale. This manipulation strategy has also been used elsewhere (Ruckmann *et al.*, 2015).

#### Stimuli

We used 5 s video clips presenting dynamic facial expressions that had been described and validated previously among another groups of subjects (Kircher *et al.*, 2013; Pohl *et al.*, 2013). Briefly, in these video clips professional actors depicted emotional facial expressions for anger and happiness. The video clips were framed with a coloured strip (either blue or green) according to the actor’s (false) identity as either a sequential or conclusive problem solver, enabling participants to make ingroup/outgroup associations.

#### fMRI stimulus setup

We used a block design with two conditions: ‘facial expression’ (happy *vs* angry) and ‘group’ (participants observing ingroup vs. outgroup, with groups coded by coloured blue or green video). Half of the actors were ingroup members, while the other half were outgroup members. In both groups, sex was equally distributed. Each condition was presented in six blocks consisting of four video clips. Each block was introduced with an instruction slide (2 s) followed by a white fixation cross (5 s) and encompassed four video clips (5 s) presenting the same facial expression. In addition, there were six blocks where only a white fixation cross was presented as a low-level baseline (25 s); these were also introduced through instruction slides (2 s). For a complete description of the paradigm, including conditions of no interest and permutation strategies, see Supplementary Material.

#### fMRI data acquisition

Imaging was conducted on a 3 T MRI scanner (MAGNETOM Trio, Siemens, Erlangen, Germany) equipped with a 12-channel head coil. Echo-planar T2*-weighted images were acquired (TR = 2250 ms, TE = 30 ms, flip angle 90°, FOV = 230 mm, distance factor = 20%) using 36 slices in an oblique axial orientation (slice order = ascending, voxel size = 3.6 mm³, interslice gap = 0.72 mm).

#### fMRI data analysis

Data were analysed using SPM8 standard routines and templates (Wellcome Department of Imaging Neuroscience, London, UK). Five initial brain volumes of each run were excluded. The remaining images were realigned to the mean image and then normalized to the standard Montreal Neurological Institute (MNI) template with volume units of 2 mm × 2 mm × 2 mm and finally smoothed with an 8 mm Gaussian filter at full-width at half maximum (FWHM). The following analyses were based on an ordinary least-squares estimation method using a general linear model. At the single-subject level, regressors were created from the time course of each condition and convoluted with a canonical hemodynamic response function. Movement parameters from realignment were integrated in the model as regressors of no interest. The low-level baseline condition was not included in the model. High-pass filtering (cut-off period of 128 s) was applied. First- and second-order time modulation was used for each condition and integrated as additional regressors in the model. For each subject, we calculated the t-contrast of the interaction (ingroup/outgroup) by emotion (happy/angry). Group analyses were performed by entering the resulting contrasts into a one-sample *t*-test (SPM8), where subjects were treated as random variables. Here, an *F*-contrast was calculated (as direction of effects were unknown) revealing the activation of the latter interaction at the group level in the ACC region of interest (ROI). In addition, this interaction was tested in an exploratory whole-brain analysis (see Supplementary Material).

ACC ROI was defined using the Wake Forest PickAtlas (www.fmri.wfubmc.edu), and the extraction and calculation of percent signal change (PSC) for each condition (ingroup happy, ingroup angry, outgroup happy and outgroup angry) was performed using the MarsBaR toolbox (http://marsbar.sourceforge.net/) for SPM8. All analyses were controlled for factors being unequally distributed among the four groups (CIT *vs* no CIT and A-allele carrier at rs1006737 *vs* no A-allele carrier at rs1006737) by including the respective information as covariates of no interest in the model (see Supplementary Material). All results were corrected for multiple comparisons at *P* < 0.05 family-wise error (FWE) on a voxel level with minimum cluster size of 50 voxels.

#### Contrasts of interest

To test the hypothesis that social categorization as either ingroup or outgroup has an effect on the neural processing of different emotional facial expressions in the ACC, we calculated an interaction analysis of the emotion (happy *vs* angry) and group membership (ingroup *vs* outgroup) factors using the ACC ROI.

Following our second hypothesis—that ACC functioning is sensitive to a gene—environment interaction between the schizophrenia risk factors rs1006737 and CIT—we used the extracted PSC for each subject and condition. GEEs with a multi-factorial design were performed in SPSS to test the 2×2×2×2 interaction of group, emotion, rs1006737 and trauma.

## Results

### MPG manipulation

In a post-fMRI debriefing, all participants correctly remembered their own problem-solver type, its associated colour and the faces of group members presented to them during scanning. Identification with the own group (ingroup) was significantly higher than with the other group (outgroup) both before and after fMRI scanning (before fMRI scanning: identification with IG = 4.93; identification with OG = 3.42; *P* < 0.001; after fMRI scanning: identification with IG = 5.21; identification with OG = 2.98; *P* < 0.001). Interestingly, the difference in identification between the ingroup and outgroup was significantly higher after scanning than before (before fMRI scanning: difference identification with IG > OG = 1.52; after fMRI scanning: difference identification with IG > OG = 2.24; *P* < 0.001), validating participants’ engagement with the fMRI task.

### Personality characteristics

The SPQ-B total score showed acceptable internal consistency (Cronbach’s α: 0.663). There was a significant interaction between the rs1006737 genotype and CIT for schizotypal personality (*P* = 0.043, Wald *χ*^2^ = 4.01), indicating that the most prominent schizotypal traits were in the group with both genetic and environmental risk factors (see Table 1). There was no main effect of rs1006737 or CIT on the SPQ-B. In a post hoc analysis, SPQ-B total score was not associated with vACC neural activity.

**Table 1.** Sample characteristics

	rs-1006737 genotype				
	GG	GG	AG/AA	AG/AA	
	no CIT	CIT	no CIT	CIT	
	(*n* = 34)	(*n*= 37)	(*n* = 55)	(*n* = 52)	*P*
Sex ratio (m/f)	22/12	12/25	32/23	24/28	0.028^a^
Age (years)	24.2 ± 3.0	24.6 ± 3.2	24.5 ± 3.7	23.2 ± 2.8	0.114^b^
SPQ-B*	3.7 ± 2.9	3.5 ± 2.6	3.2 ± 2.5	4.7 ± 3.0	0.038^b^
THQ	5.6 ± 6.08	8.8 ± 8.7	6.1 ± 6.0	8.7 ± 6.0	0.040^b^
CIT	–	2.1 ± 1.3	–	1.75 ± 0.8	0.208^c^

Note: Values represent mean ± standard deviation; SPQ-B, Schizotypal Personality Questionnaire-Brief; THQ, Trauma History Questionnaire; CIT, Childhood Interpersonal Trauma.

a Pearson χ^2^ for differences in frequencies.

b One-way ANOVA for mean differences.

c Two-sample t-test.

* *P* = <0. 05 for interaction of rs1006737 (GG *vs* GA/AA) and childhood interpersonal trauma (yes *vs* no).

### fMRI results

**Fig. 1.**
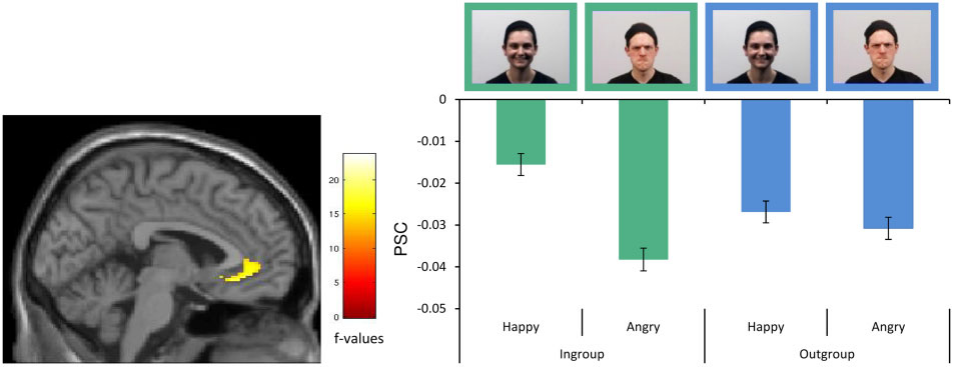
Interaction of Emotion and Group. The interaction of group (ingroup *vs* outgroup) and emotion (happy *vs* angry) factors in the ACC ROI is driven by differences in emotional ingroup processing. PSC = percent signal change. Sex was equally distributed among the ingroup and outgroup and all actors depicted all facial expressions.

In the ACC ROI analyses, we found a significant interaction between the MPG group (ingroup/outgroup) and emotion (happy/angry), suggesting that group membership influences the processing of facial emotions in the vACC (MNI xyz = 8, 42, 0; F = 23.71; cluster size k = 423 voxels; df = 177; *P*<0.001; FWE corrected). This interaction effect was confirmed by exploratory whole-brain analyses (see Supplementary Figure S1 and Supplementary Table S1). The bar graphs in Figure 1 illustrate the PSC for activity in the ACC ROI and indicate that emotional valence modulated vACC activation for ingroup members only—there was a stronger deactivation for angry than for happy facial ingroup emotion. There were no significant main effects of MPG group membership or emotional facial expression in the ACC ROI. For the respective results of the whole-brain analyses, see Supplementary Material.

### Gene–environment interaction

**Fig. 2.**
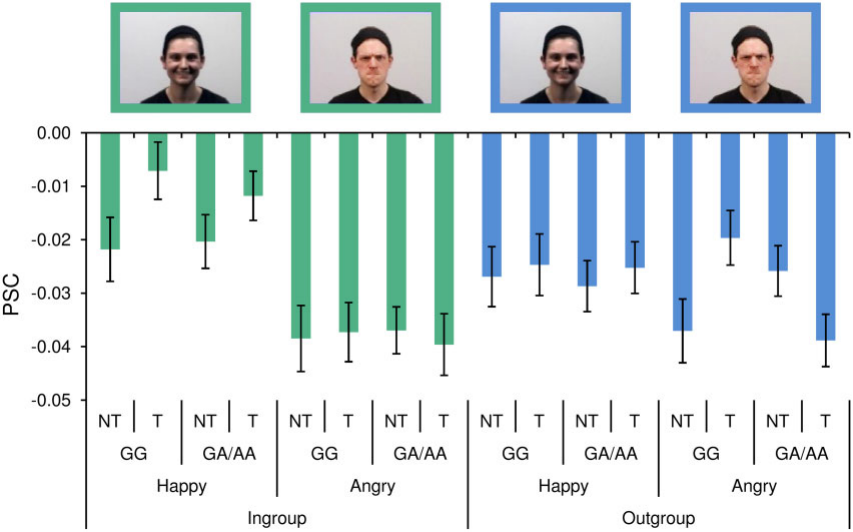
Interaction of rs1006737 and childhood trauma with emotion and group. At the condition level, there is significant interaction between rs1006737 and trauma, particularly for processing angry outgroup emotions. T = childhood trauma; NT = no childhood trauma; GG or GA/AA of rs1006737 (A=risk allele); PSC = percent signal change. Sex was equally distributed among the ingroup and outgroup and all actors depicted all facial expressions.

There was a significant interaction for neural processing within the vACC between the factors rs1006737 (GG *vs* GA/AA), CIT (yes *vs* no), emotional valence (happy *vs* angry) and group membership (ingroup *vs* outgroup; Wald *χ*^2^ = 4.753, *P* < 0.05; see Figure 2). The bar graphs in Figure 2 illustrate the PSC for the activity in the vACC for the 16 conditions (2×2×2×2). There was a main effect of trauma for the processing of happy ingroup members (green bars on the far left; post-hoc interaction Wald *χ*^2^ = 5.2, *P* < 0.05). Most interestingly, there was an interaction between rs1006737 and trauma for vACC activity in response to the angry outgroup (blue bars on the far right; post-hoc interaction Wald *χ*^2^ = 9.1, *P* < 0.01; see Figure 2). Importantly, these results remained significant when using a continuous variable for CIT (see Supplementary Material). We found no main effects for rs1006737 and CIT in terms of vACC activation across conditions (for both, *P* > 0.2) and no effect of genetic and environmental risk factors for facial expressions in the angry ingroup or happy outgroup (for all, *P* > 0.2).

## Discussion

Genetic and environmental factors contribute to the development of schizophrenia. However, the specific neural pathways and interactions of different risk factors remain to be further clarified (Van Os *et al.*, 2014). Here, we investigated the neural correlates of ingroup vs. outgroup processing in a large group of healthy subjects using fMRI by applying a MGP with emotional facial expressions as video stimuli. In line with our hypotheses, we found (1) an interaction of neural activation for the processing of emotional valence (happy *vs* angry) and group membership (ingroup *vs* outgroup) in the vACC. We further revealed (2) an interaction of vACC activation during group-dependent (ingroup *vs* outgroup) emotion processing for the risk factors CIT and rs1006737. Specifically for angry outgroup emotion processing, there were different effects of trauma depending on genetic predisposition.

The interaction of the two factors, emotional valence and group membership, was driven by differences in vACC activation in terms of the emotional valence of a presented face (angry or happy) for encounters with ingroup members only. This was reflected by a stronger deactivation for angry compared to happy facial expressions for ingroup members. The vACC is a key region in the fronto-limbic emotion regulatory system (Etkin *et al.*, 2011). A distinction of the emotional valence on a neural level in this region might reflect a higher emotional relevance of the ingroup. Indeed, previously the vACC has been found to be activated during the processing of self-related stimuli (Heatherton, 2011). However, at the same time, reduced emotional discrimination for outgroup members in this region also reflects an indifference for opposite emotional signals of outgroup members on a neural level, which might become relevant in situations where confrontation with outgroup members is frequent—as in urban dwellers or migrants. Indeed, misperception of emotions has been shown for faces not belonging to the subject’s own race (Elfenbein and Ambady, 2002). However, here we showed for the first time similar results on a neural level using a MGP and therefore, we experimentally avoided ethnic bias. Such an indifference regarding outgroup emotional signals might also have consequences on a behavioural level. In the study by Volz and colleagues, neural vACC activation has been associated with altered behaviour towards the (minimal) outgroup in terms of a discrimination of the outgroup in a money distribution game (Volz and Kessler, 2009). Hence, altered vACC activation regarding group membership is related to an intergroup bias in both processing of emotional signals and behavior.

With our study, we were able to show that the processing of ingroup/outgroup emotional signals in the vACC furthermore interacts with the genetic and environmental risk factors rs1006737 and CIT, respectively. We found a significant four-fold interaction of the (within-subject) factors group and emotion and the (between-subject) factors rs1006737 and CIT. This was driven by a significant interaction of rs1006737 and CIT specifically for angry outgroup emotions. Depending on rs1006737 risk allele carrier status (GG *vs* GA/AA) CIT was related to an increased (GG) or decreased (GA/AA) vACC activation during angry outgroup processing (see Figure 2). This inverted effect of CIT results in a similar vACC activation for angry outgroup facial expressions in subjects with no risk factors (GG + NT) and the subjects with both risk factors (GA/AA + T). Hence, we cannot interpret this interaction as a simple combined effect of both risk factors. Regarding the vACC in its interplay with the limbic system, CIT and rs1006737 both are related to alterations on various levels. Functional fronto-limbic connectivity for emotional facial expressions is reduced in individuals carrying the rs1006737 risk allele (Wang *et al.*, 2011) and altered in a complex manner in individuals with aversive childhood experience (Williams *et al.*, 2006). On a brain structural level, CIT and rs1006737 have opposite effects in the vACC: rs1006737 risk allele is associated with increased vACC volume (Wang *et al.*, 2011) while childhood emotional maltreatment and traumatic experiences are associated with reduced vACC volume (Cohen *et al.*, 2006; Van Harmelen *et al.*, 2010; Baker *et al.*, 2013). Increased vACC volume in rs1006737 risk allele carriers might reflect a compensatory mechanism of inefficient neuroplasticity (Wang *et al.*, 2011) whilst CIT negatively affects neurogenesis as well as production of neurotrophic factors through a stress-induced release of glucocorticoids and pro-inflammatory cytokines (Cattaneo *et al.*, 2015). A history of trauma induces changes and aberrant functioning of the hypothalamic pituitary-adrenal axis (Miller *et al.*, 2007) which is associated with depression or schizophrenia and other psychotic disorders (Krishnan and Nestler, 2008; Walker *et al.*, 2008). At the same time L-type voltage-gated calcium channels in the limbic system encoded by *CACNA1C* are involved in the regulation of the hypothalamic pituitary-adrenal axis by exhibiting glucocorticoid-mediated increases in calcium currents (Karst *et al.*, 2002; Chameau *et al.*, 2007). Furthermore, given the effects of rs1006737 on neuroplasticity and memory formation in frontal and mesolimbic structures (Erk *et al.*, 2010; Wang *et al.*, 2011; Bhat *et al.*, 2012), trauma and rs1006737 are likely to converge in brain structure and functioning on multiple levels in a complex manner. Hence, the inverted effect in vACC activation of CIT depending on rs1006737 risk allele carrier status for angry outgroup processing, revealed by our study, is likely to be a result of a complex interaction in neuroplasticity and connectivity of the vACC and converging effects of both risk factors in associated brain regions. Future studies have to disentangle the complex network(s) in which the inverted effect by CIT on angry outgroup emotion processing is embedded. Our results substantiate that relevant effects of genetic and environmental (risk) factors on a neural level evolve through their interaction and remain hidden when examining them in isolation. However, with this study, we could for the first time reveal an interaction of gene (rs1006737) and environment (CIT) in vACC ingroup/outgroup processing.

Ingroup/outgroup processing is especially relevant for individuals moving in complex social structures with a variety of groups—such as urban dwellers and migrants. Indeed, urban dwellers and migrants both show relevant alterations in vACC functioning—urban living and upbringing has been associated with differences in vACC functioning in response to social evaluative stress (Lederbogen *et al.*, 2011). An ethnic minority group exhibited increased heart rate and increased subjective emotional response as well as differences in vACC functioning and connectivity in response to social stress compared to an autochthon control group (Akdeniz *et al.*, 2014b). Another study revealed a reduced vACC volume and, in addition, a negative correlation of early urbanicity and vACC volume in male migrants (Akdeniz *et al.*, 2017). The causal influence of migration and urban living on schizophrenia risk is not yet fully understood (Cantor-Graae and Pedersen, 2013; Heinz *et al.*, 2013; Van Os *et al.*, 2014). Based on our results, we speculate that the ubiquitous encounter of outgroup members, experienced by migrants and urban dwellers, is—in combination with genetic and additional environmental risk—in itself a risk factor for schizophrenia (Abed and Abbas, 2011).

An incidental finding was the association of CIT with a lower vACC deactivation for happy ingroup facial expressions resulting in an increased difference between neural activation for happy and angry ingroup processing. In the past, childhood maltreatment was related to an increased difference between vACC activation for happy and sad facial expressions (Dannlowski *et al.*, 2013). Furthermore, childhood maltreatment is associated with an attention bias away from happy facial emotions depending on attachment style (Davis *et al.*, 2014). However, our finding implies an intergroup bias in this context, which should be further investigated in the future.

The subgroup with combined risk, presence of rs1006737 risk allele and experience of CIT (GA/AA + T), showed higher schizotypal traits than the other groups. This finding shows that in our sample the interaction of rs1006737 and CIT is in fact associated with higher schizophrenia vulnerability (Vollema *et al.*, 2002). Importantly, schizotypal traits were not associated with vACC functioning, indicating that the effects described above are not confounded by variability in schizotypal traits in our sample but can be traced back to the interaction of rs1006737 and CIT.

Several limitations of this study have to be noted. During fMRI scanning, the participants only watched videos and no behavioral data, self-report data or physiological measures were collected to not distort the automatic response to the presented faces. Hence, there are no additional data from the scanner with which to correlate our imaging results. Further, although happy and angry facial expressions represent two contrary emotions, there are plenty of other emotions and nuances of facial expressions.

With this study, we demonstrate that distinction of opposite emotional signals regarding neural activity in the vACC—a major hub of emotional processing and regulation—depends on group membership. Furthermore, we confirm the putative role of the vACC as a converging zone of genetic and environmental risk factors of schizophrenia by demonstrating the modulation of vACC functioning in response to negative outgroup emotion by childhood interpersonal trauma and rs1006737 risk allele carrier status. On a neurophysiological basis, gene–environment interactions are still poorly understood and the investigation of these effects is in its early stages (Van Os *et al.*, 2014). There is huge potential in this research area for increasing our understanding of the pathogenesis of psychiatric disorders. With our work, we took a step in this direction by revealing for the first time that a gene–environment interaction of schizophrenia risk factors influences facial emotion processing depending on group membership.

## Supplementary Material

Supplementary Data

## References

[nsy004-B1] Abed R.T., Abbas M.J. (2011). A reformulation of the social brain theory for schizophrenia: the case for out-group intolerance. Perspectives in Biology and Medicine, 54, 132–51. 21532129 10.1353/pbm.2011.0020

[nsy004-B2] Adams R.B., Rule N.O., Franklin R.G., Wang E., Stevenson M.T., Yoshikawa S. (2010). Cross-cultural reading the mind in the eyes: an fMRI investigation. Journal of Cognitive Neuroscience, 22(1), 97–108. 19199419 10.1162/jocn.2009.21187

[nsy004-B3] Akdeniz C., Schäfer A., Streit F., (2017) Sex-dependent association of perigenual anterior cingulate cortex volume and migration background, an environmental risk factor for Schizophrenia. Schizophrenia Bulletin, 43 *(*4*)*, 925–34. 28969352 10.1093/schbul/sbw138 PMC5472165

[nsy004-B4] Akdeniz C., Tost H., Meyer-Lindenberg A. (2014a). The neurobiology of social environmental risk for schizophrenia: an evolving research field. Social Psychiatry and Psychiatric Epidemiology, 49, 507–17. 24638893 10.1007/s00127-014-0858-4

[nsy004-B5] Akdeniz C., Tost H., Streit F., (2014b). Neuroimaging evidence for a role of neural social stress processing in ethnic minority-associated environmental risk. JAMA Psychiatry, 71(6), 672–80. 24740491 10.1001/jamapsychiatry.2014.35

[nsy004-B6] Baker L.M., Williams L.M., Korgaonkar M.S., Cohen R.A., Heaps J.M., Paul R.H. (2013). Impact of early vs. late childhood early life stress on brain morphometrics. Brain Imaging and Behavior, 7, 196–203. 23247614 10.1007/s11682-012-9215-y PMC8754232

[nsy004-B7] Bhat S., Dao D.T., Terrillion C.E., (2012). CACNA1C (Cav1.2) in the pathophysiology of psychiatric disease. Progress in Neurobiology, 99(1), 1–14. 22705413 10.1016/j.pneurobio.2012.06.001 PMC3459072

[nsy004-B8] Cantor-Graae E., Pedersen C.B. (2013). Full spectrum of psychiatric disorders related to foreign migration: a Danish population-based cohort study. JAMA Psychiatry, 70(4), 427–35. 23446644 10.1001/jamapsychiatry.2013.441

[nsy004-B9] Cantor-Graae E., Selten J.P. (2005). Schizophrenia and migration: a meta-analysis and review. American Journal of Psychiatry, 162, 12–24. 15625195 10.1176/appi.ajp.162.1.12

[nsy004-B10] Cattaneo A., Macchi F., Plazzotta G., (2015). Inflammation and neuronal plasticity: a link between childhood trauma and depression pathogenesis. Frontiers in Cellular Neuroscience, 9, 1–12. 25873859 10.3389/fncel.2015.00040 PMC4379909

[nsy004-B11] Chameau P., Qin Y., Spijker S., Smit G., Joels M. (2007). Glucocorticoids specifically enhance L-type calcium current amplitude and affect calcium channel subunit expression in the mouse hippocampus. Journal of Neurophysiology, 97(1), 5–14. 17021021 10.1152/jn.00821.2006

[nsy004-B12] Cikara M., Van Bavel J.J. (2014). The neuroscience of intergroup relations: an integrative review. Perspectives on Psychological Science, 9(3), 245–74. 26173262 10.1177/1745691614527464

[nsy004-B13] Cohen R. a., Grieve S., Hoth K.F., (2006). Early life stress and morphometry of the adult anterior cingulate cortex and caudate nuclei. Biological Psychiatry, 59(10), 975–82. 16616722 10.1016/j.biopsych.2005.12.016

[nsy004-B14] Dannlowski U., Kugel H., Huber F., (2013). Childhood maltreatment is associated with an automatic negative emotion processing bias in the amygdala. Human Brain Mapping, 34(11), 2899–909. 22696400 10.1002/hbm.22112 PMC6870128

[nsy004-B15] Dannlowski U., Stuhrmann A., Beutelmann V., (2012). Limbic scars: long-term consequences of childhood maltreatment revealed by functional and structural magnetic resonance imaging. Biological Psychiatry, 71(4), 286–93. 22112927 10.1016/j.biopsych.2011.10.021

[nsy004-B16] Das-Munshi J., Bécares L., Boydell J.E., (2012). Ethnic density as a buffer for psychotic experiences: findings from a national survey (EMPIRIC). British Journal of Psychiatry, 201(04), 282–90. 22844021 10.1192/bjp.bp.111.102376 PMC3461446

[nsy004-B17] Davis J.S., Fani N., Ressler K., Jovanovic T., Tone E.B., Bradley B. (2014). Attachment anxiety moderates the relationship between childhood maltreatment and attention bias for emotion in adults. Psychiatry Research, 217, 79–85. 24680873 10.1016/j.psychres.2014.03.010 PMC4060600

[nsy004-B18] Diehl M. (1990). The minimal group paradigm: theoretical explanations and empirical findings. European Review of Social Psychology, 1(1), 263–92.

[nsy004-B19] Dovidio J.F., Hebl M., Richeson J. a., Shelton J.N. (2006). Nonverbal communication, race, and intergroup interaction. In: Manusov, V.L., Patterson, M.L. (ed.). *The Sage Handbook of Nonverbal Communication*, Thousand Oaks, CA: Sage, pp. 481–500.

[nsy004-B20] Elfenbein H.A., Ambady N. (2002). On the universality and cultural specificity of emotion recognition: a meta-analysis. Psychological Bulletin, 128, 203–35. 11931516 10.1037/0033-2909.128.2.203

[nsy004-B21] Eres R., Molenberghs P. (2013). The influence of group membership on the neural correlates involved in empathy. Frontiers in Human Neuroscience, 7, 176. 23653604 10.3389/fnhum.2013.00176 PMC3644680

[nsy004-B22] Erk S., Meyer-Lindenberg A., Schmierer P., (2014). Hippocampal and frontolimbic function as intermediate phenotype for psychosis: evidence from healthy relatives and a common risk variant in CACNA1C. Biological Psychiatry, 76(6), 466–75. 24411473 10.1016/j.biopsych.2013.11.025

[nsy004-B23] Erk S., Meyer-Lindenberg A., Schnell K., (2010). Brain function in carriers of a genome-wide supported bipolar disorder variant. Archives of General Psychiatry, 67(8), 803–11. 20679588 10.1001/archgenpsychiatry.2010.94

[nsy004-B24] Etkin A., Egner T., Kalisch R. (2011). Emotional processing in anterior cingulate and medial prefrontal cortex. Trends in Cognitive Sciences, 15, 85–93. 21167765 10.1016/j.tics.2010.11.004 PMC3035157

[nsy004-B25] Ferreira M.A.R., O'Donovan M.C., Meng Y.A., (2008). Collaborative genome-wide association analysis supports a role for ANK3 and CACNA1C in bipolar disorder. Nature Genetics 40(9), 1056–8. 18711365 10.1038/ng.209 PMC2703780

[nsy004-B26] Fusar-Poli P., Smieskova R., Kempton M.J., Ho B.C., Andreasen N.C., Borgwardt S. (2013). Progressive brain changes in schizophrenia related to antipsychotic treatment? A meta-analysis of longitudinal MRI studies. Neuroscience and Biobehavioral Reviews, 37, 1680–91. 23769814 10.1016/j.neubiorev.2013.06.001 PMC3964856

[nsy004-B27] Gilbert R., Widom C.S., Browne K., Fergusson D., Webb E., Janson S. (2009). Burden and consequences of child maltreatment in high-income countries. The Lancet, 373, 68–81. 10.1016/S0140-6736(08)61706-7 19056114

[nsy004-B28] Green B. (1996). Trauma History Questionnaire In: Stamm B.H., (ed.). Measurement of Stress, Trauma, and Adaptation, Lutherville, Md: Sidran Press, pp. 366–9.

[nsy004-B29] Green E.K., Grozeva D., Jones I., (2010). The bipolar disorder risk allele at CACNA1C also confers risk of recurrent major depression and of schizophrenia. Molecular Psychiatry, 15(10), 1016–22. 19621016 10.1038/mp.2009.49 PMC3011210

[nsy004-B30] Heatherton T.F. (2011). Neuroscience of self and self-regulation. Annual Review of Psychology, 62, 363–90. 10.1146/annurev.psych.121208.131616 PMC3056504 21126181

[nsy004-B31] Heinz A., Deserno L., Reininghaus U. (2013). Urbanicity, social adversity and psychosis. World Psychiatry, 12(3), 187–97. 24096775 10.1002/wps.20056 PMC3799240

[nsy004-B32] Hugenberg K., Bodenhausen G.V. (2004). Ambiguity in social categorization: the role of prejudice and facial affect in race categorization. Psychological Science, 15, 342–5. 15102145 10.1111/j.0956-7976.2004.00680.x

[nsy004-B33] Karst H., Nair S., Velzing E., (2002). Glucocorticoids alter calcium conductances and calcium channel subunit expression in basolateral amygdala neurons. European Journal of Neuroscience, 16(6), 1083–9. 12383237 10.1046/j.1460-9568.2002.02172.x

[nsy004-B34] Kircher T., Pohl A., Krach S., (2013). Affect-specific activation of shared networks for perception and execution of facial expressions. Social Cognitive and Affective Neuroscience, 8(4), 370–7. 22275167 10.1093/scan/nss008 PMC3624947

[nsy004-B35] Krishnan V., Nestler E.J. (2008). The molecular neurobiology of depression. Nature, 455(7215), 894–902. 18923511 10.1038/nature07455 PMC2721780

[nsy004-B36] Lederbogen F., Kirsch P., Haddad L., (2011). City living and urban upbringing affect neural social stress processing in humans. Nature, 474(7352), 498–501. 21697947 10.1038/nature10190

[nsy004-B37] Matheson S.L., Shepherd A.M., Pinchbeck R.M., Laurens K.R., Carr V.J. (2012). Childhood adversity in schizophrenia: a systematic meta-analysis. Psychological Medicine, 43, 1–13. 22716913 10.1017/S0033291712000785

[nsy004-B38] Miller G.E., Chen E., Zhou E.S. (2007). If it goes up, must it come down? Chronic stress and the hypothalamic-pituitary- adrenocortical axis in humans. Psychological Bulletin, 133, 25–45. 17201569 10.1037/0033-2909.133.1.25

[nsy004-B39] New A.S., Fan J., Murrough J.W., (2009). A functional magnetic resonance imaging study of deliberate emotion regulation in resilience and posttraumatic stress disorder. Biological Psychiatry, 66(7), 656–64. 19589502 10.1016/j.biopsych.2009.05.020

[nsy004-B40] Nyegaard M., Demontis D., Foldager L., (2010). CACNA1C (rs1006737) is associated with schizophrenia. Molecular Psychiatry, 15(2), 119–21. 20098439 10.1038/mp.2009.69

[nsy004-B41] Pohl A., Anders S., Schulte-Rüther M., Mathiak K., Kircher T. (2013). Positive facial affect—an fMRI study on the involvement of insula and amygdala. PLoS One, 8, e69886. 10.1371/journal.pone.0069886 PMC3749202 23990890

[nsy004-B42] Raine A., Benishay D. (1995). The SPQ-B: a brief screening instrument for schizotypal personality disorder. Journal of Personality Disorders, 9, 346–55.

[nsy004-B43] Rao S., Yao Y., Zheng C., (2016). Common variants in CACNA1C and MDD susceptibility: a comprehensive meta-analysis. American Journal of Medical Genetics, Part B: Neuropsychiatric Genetics, 171(6), 896–903. 10.1002/ajmg.b.32466 27260792

[nsy004-B44] Ripke S., Neale B.M., Corvin A., (2014). Biological insights from 108 schizophrenia-associated genetic loci. Nature, 511(7510), 421–7. 25056061 10.1038/nature13595 PMC4112379

[nsy004-B45] Ripke S., O'Dushlaine C., Chambert K., (2013). Genome-wide association analysis identifies 13 new risk loci for schizophrenia. Nature Genetics, 45(10), 1150–9. 23974872 10.1038/ng.2742 PMC3827979

[nsy004-B46] Roussos P., Bitsios P., Giakoumaki S.G., (2013). CACNA1C as a risk factor for schizotypal personality disorder and schizotypy in healthy individuals. Psychiatry Research, 206(1), 122–3. 22985546 10.1016/j.psychres.2012.08.039 PMC4176879

[nsy004-B47] Ruckmann J., Bodden M., Jansen A., Kircher T., Dodel R., Rief W. (2015). How pain empathy depends on ingroup/outgroup decisions: a functional magnet resonance imaging study. Psychiatry Research: Neuroimaging, 234, 1–9. 26323252 10.1016/j.pscychresns.2015.08.006

[nsy004-B48] Sklar P., Smoller J.W., Fan J., (2008). Whole-genome association study of bipolar disorder. Molecular Psychiatry, 13(6), 558–69. 18317468 10.1038/sj.mp.4002151 PMC3777816

[nsy004-B49] Starnawska A., Demontis D., Pen A., (2016). CACNA1C hypermethylation is associated with bipolar disorder. Translational Psychiatry, 6(6),e831. 27271857 10.1038/tp.2016.99 PMC4931616

[nsy004-B50] Tajfel H. (1970). Experiments in intergroup discrimination. Scientific American, 223(5), 96–102. 5482577

[nsy004-B51] Tajfel H., Billig M.G., Bundy R.P., Flament C. (1971). Social categorization and intergroup behaviour. European Journal of Social Psychology, 1, 149–78.

[nsy004-B52] Takahashi S., Glatt S.J., Uchiyama M., Faraone S.V., Tsuang M.T. (2015). Meta-analysis of data from the Psychiatric Genomics Consortium and additional samples supports association of CACNA1C with risk for schizophrenia. Schizophrenia Research, 168, 429–33. 26276307 10.1016/j.schres.2015.07.033

[nsy004-B53] Taylor S.F., Kang J., Brege I.S., Tso I.F., Hosanagar A., Johnson T.D. (2012). Meta-analysis of functional neuroimaging studies of emotion perception and experience in schizophrenia. Biological Psychiatry, 71, 136–45. 21993193 10.1016/j.biopsych.2011.09.007 PMC3237865

[nsy004-B54] Tost H., Meyer-Lindenberg A. (2012). Puzzling over schizophrenia: schizophrenia, social environment and the brain. Nature Medicine, 18, 211–3. 10.1038/nm.2671 22310688

[nsy004-B55] Van Harmelen A.L., Van Tol M.J., Van Der Wee N.J., (2010). Reduced medial prefrontal cortex volume in adults reporting childhood emotional maltreatment. Biological Psychiatry, 68(9), 832–8. 20692648 10.1016/j.biopsych.2010.06.011

[nsy004-B56] Van Os J., Rutten B.P., Myin-Germeys I., (2014). Identifying gene-environment interactions in schizophrenia: contemporary challenges for integrated, large-scale investigations. Schizophrenia Bulletin, 40(4), 729–36. 24860087 10.1093/schbul/sbu069 PMC4059449

[nsy004-B57] Vassos E., Pedersen C.B., Murray R.M., Collier D. a., Lewis C.M. (2012). Meta-analysis of the association of urbanicity with schizophrenia. Schizophrenia Bulletin, 38, 1118–23. 23015685 10.1093/schbul/sbs096 PMC3494055

[nsy004-B58] Vollema M.G., Sitskoorn M.M., Appels M.C.M., Kahn R.S. (2002). Does the Schizotypal Personality Questionnaire reflect the biological-genetic vulnerability to schizophrenia? Schizophrenia Research, 54, 39–45. 11853977 10.1016/s0920-9964(01)00350-4

[nsy004-B59] Volz K.G., Kessler T. (2009). In-group as part of the self : in-group favoritism is mediated by medial prefrontal cortex activation. Social Neuroscience, 4, 244–60. 19085561 10.1080/17470910802553565

[nsy004-B60] Walker E., Mittal V., Tessner K. (2008). Stress and the hypothalamic pituitary adrenal axis in the developmental course of schizophrenia. Annual Review of Clinical Psychology, 4, 189–216. 10.1146/annurev.clinpsy.4.022007.141248 18370616

[nsy004-B61] Wang F., Mcintosh A.M., He Y., Gelernter J., Blumberg H.P. (2011). The association of genetic variation in CACNA1C with structure and function of a frontotemporal system. Bipolar Disorders, 13, 696–700. 22085483 10.1111/j.1399-5618.2011.00963.x PMC3233238

[nsy004-B62] Wheeler M.E., Fiske S.T. (2005). Controlling racial prejudice social-cognitive goals affect amygdala and stereotype activation. Psychological Science, 16, 56–63. 15660852 10.1111/j.0956-7976.2005.00780.x

[nsy004-B63] Williams L.M., Kemp A.H., Felmingham K., (2006). Trauma modulates amygdala and medial prefrontal responses to consciously attended fear. NeuroImage, 29(2), 347–57. 16216534 10.1016/j.neuroimage.2005.03.047

