# Supplementary material for: Outgroup emotion processing in the vACC is modulated by childhood trauma and CACNA1C risk variant
Source: Soc Cogn Affect Neurosci. 2018 Jan 29;13(3):341–8. doi: 10.1093/scan/nsy004 (PMC5836282; doi:10.1093/scan/nsy004)
Supplement: Supplementary Data [file nsy004_supp.pdf]

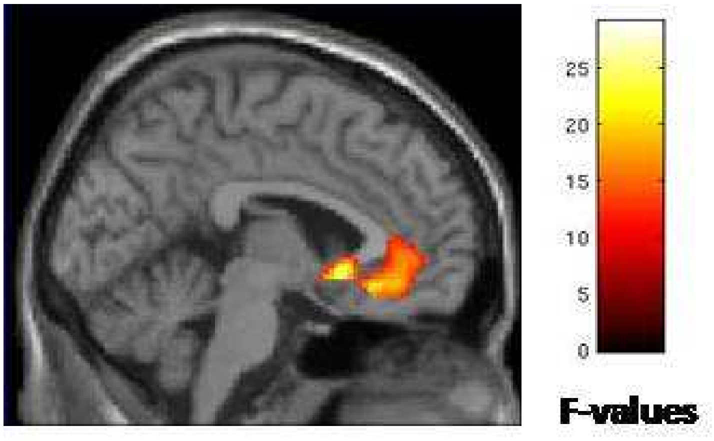

Supplementary Figure 1

Activation of the interaction between the factors emotion and group (whole-brain analysis, p<0.001 uncorr., minimum cluster size 50 voxels).

Supplementary Table 1 Number of childhood interpersonal traumatic events (18 and younger)

Interpersonal traumatic events	0	1	2	3	4	5	6
Subjects	68	48	32	20	6	3	1

Supplementary Table 2 Regions activated by the interaction between the factors emotion and group (whole-brain analysis, p<0.001 uncorr., minimum cluster size 50 voxels).

Brain region	f-value	x	y	z	size
**Right superior frontal gyrus**	**29.06**	**16**	**48**	**0**	**2 610**
Left caudate nucleus	24.88	-4	18	-6	X
Right anterior cingulate	23.71	8	42	0	X
**Left putamen**	**15.71**	**-24**	**6**	**10**	**102**
Left caudate nucleus	11.98	-24	18	4	X

Supplementary Table 3 Regions activated by the emotion main effect (whole-brain analysis, p<0.001 uncorr., minimum cluster size 50 voxels).

Brain region	f-value	x	y	z	size
**Left middle occipital gyrus**	**132.99**	**-22**	**-100**	**4**	**6 969**
Right middle occipital gyrus	115.97	26	-100	6	X
Right calcarine gyrus	47.49	8	-86	8	X
**Left inferior parietal lobule**	**102.36**	**-34**	**-48**	**44**	**13 046**
Left middle temporal gyrus	65.53	-46	-54	8	X
Left superior parietal lobule	59.68	-22	-70	60	X
**Left precentral gyrus**	**80.52**	**-48**	**8**	**34**	**8 769**
Left inferior frontal gyrus, pars orbicularis	74.65	-42	38	-10	X
Left precentral gyrus	62.02	-48	6	20	X
**Left superior medial frontal gyrus**	**59.43**	**-14**	**52**	**12**	**5 759**
Right superior medial frontal gyrus	52.78	14	54	14	X
Right middle frontal gyrus, pars orbitalis	46.34	6	40	-10	X
**Right cerebellum**	**39.82**	**20**	**-76**	**-48**	**915**

Right cerebellum	34.45	14	-80	-36	X
Right cerebellum	19.7	32	-68	-50	X
**Right precentral gyrus**	**38.8**	**50**	**10**	**34**	**2 494**
Right frontal inferior gyrus, pars triangularis	36.23	54	34	0	X
Right frontal inferior gyrus, pars opercularis	34.84	46	8	26	X
**Left cerebellum**	**37.48**	**-12**	**-80**	**-34**	**706**
Left cerebellum	21.85	-28	-72	-50	X
**Right middle cingulate cortex**	**33**	**0**	**-28**	**26**	**405**
Left middle cingulate cortex	18.95	-8	-26	38	X
**Right inferior temporal gyrus**	**25.07**	**50**	**-46**	**-16**	**81**
**Left superior temporal gyrus**	**19.88**	**-64**	**-28**	**8**	**197**
Left supramarginal gyrus	16.99	-42	-30	24	X
**Left thalamus**	**19.54**	**-8**	**-12**	**6**	**249**
Left thalamus	15.09	-16	-26	8	X
Left caudate nucleus	12.21	-14	-4	14	X
**Right superior temporal gyrus**	**18.92**	**62**	**-24**	**8**	**55**
**Right middle temporal gyrus**	**17.41**	**58**	**-52**	**2**	**281**
Right middle temporal gyrus	17.39	44	-56	10	X
Right middle temporal gyrus	15.49	50	-52	6	X

Supplementary Table 4 Regions activated by the group main effect (whole-brain analysis, p<0.001 uncorr., minimum cluster size 50 voxels).

**Brain region**	**f-value**	**x**	**y**	**z**	**size**
**Right frontal middle gyrus**	**28.24**	**46**	**44**	**18**	**1 384**
Right frontal middle gyrus	24.85	42	34	36	X
Right frontal middle gyrus, pars orbitalis	24.29	34	54	-2	X
**Left superior frontal gyrus**	**23.31**	**-16**	**52**	**10**	**177**
Left middle frontal gyrus	21.19	-32	54	8	X

**Left superior temporal gyrus**	**23.31**	**-42**	**0**	**-12**	**198**
Right superior parietal lobule	**21.18**	**50**	**-42**	**60**	**847**
Right inferior parietal lobule	20.05	54	-32	50	X
Right superior parietal lobule	19.66	38	-52	58	X
**Left superior occipital lobule**	**21.06**	**-10**	**-94**	**2**	**844**
Right calcarine gyrus	16.55	10	-96	2	X
Right calcarine gyrus	16.5	16	-88	-2	X
Left inferior frontal gyrus, pars triangularis	19.84	-36	38	10	50
**Right insula**	**16.83**	**42**	**6**	**-12**	**119**
**Right cerebellum**	**15.37**	**24**	**-46**	**-32**	**77**
Right cerebellum	12.49	24	-54	-30	X
**Right cerebellum**	**3.65**	**-14**	**-80**	**-16**	**98**
**Right angular gyrus**	**13.49**	**28**	**-58**	**44**	**117**
Right superior parietal lobule	13.42	22	-64	58	X
